# A higher incidence of smooth endoplasmic reticulum clusters with aromatase inhibitors

**DOI:** 10.1002/rmb2.12296

**Published:** 2019-09-11

**Authors:** Hiroe Saito, Junko Otsuki, Hiromi Takahashi, Rei Hirata, Toshihiro Habara, Nobuyoshi Hayashi

**Affiliations:** ^1^ Okayama Couple’s Clinic Okayama Japan; ^2^ Assisted Reproductive Technology Center Okayama University Okayama Japan

**Keywords:** aromatase inhibitor, clomiphene citrate, estradiol, progesterone, smooth endoplasmic reticulum

## Abstract

**Purpose:**

This study aimed to analyze whether a regimen of aromatase inhibitor (AI) could reduce the occurrence of smooth endoplasmic reticulum clusters (sERCs) in oocytes.

**Method(s):**

The AI and the clomiphene citrate (CC) regimens were compared, regarding the sERC (+) rates and the serum estradiol and progesterone levels on the date of hCG administration, and the duration of AI, CC, and hMG administration.

**Result(s):**

The occurrence of sERCs in oocytes from patients treated with AI was significantly higher than that in oocytes from those treated with CC. Both the serum estradiol and progesterone levels were found to be significantly higher in sERC (+) than in sERC (‐) cycles. With regard to the CC cycles, no significant differences were detected. The duration of AI and hMG administration was longer for sERC (+) than for sERC (‐) cycles.

**Conclusion:**

As AI did not reduce the occurrence of sERCs, the elevation of estradiol may not be the cause of sERC occurrence but a consequence. Considering the higher levels of progesterone and longer duration of hMG in sERC (+) cycles, the negative effects of premature luteinization, which frequently occur with the AI protocol, should be investigated further.

## 1 INTRODUCTION

Smooth endoplasmic reticulum clusters (sERCs) have been known to appear occasionally in human oocytes. A cumulative number of abnormalities in live births, following the transfer of embryos from sERC (+) cycles and oocytes, has been reported,^1^, ^2^, ^3^, ^4^, ^5^ although healthy babies can be born from embryos derived from sERC (+) oocytes.^6^, ^7^ The clinical impact of sERC‐positive oocytes remains controversial; however, two recent studies reported that sERC‐positive oocytes had negative biological impact. Canto et al reported that sERC‐positive oocytes were prone to a higher frequency of aberrant spindle formation. Furthermore, sERC‐positive oocytes have been strongly associated with aberrant actin distribution in their sub‐oolemmal regions.^8^ Otsuki et al reported that the incidence of meiotic and mitotic cytokinesis failure was higher in embryos derived from sERC‐positive oocytes than in embryos derived from sERC‐negative oocytes.^9^ An embryonic cell that experiences cytokinesis failure during meiosis results in 3PN, which during mitosis could become tetraploid and might cause abnormal chromosomal configurations in the embryo.^9^ However, the mechanism underlying sERC formation is still unknown. A high concentration of estradiol per oocyte when ovulation is triggered may be an indicator of sERC predisposition,^1^ as it is well known that estradiol levels increase in proportion to the growth of ovarian follicles. It has also been reported that sERC formation is related to the higher serum estradiol 1, 2, 10 and progesterone 11 levels detected in sERC‐positive cycles.

The enzyme aromatase synthesizes estrogen, and aromatase inhibitor (AI) suppresses the production of estrogen.^12^ Ovarian stimulation with AI has been proposed as one of the treatments for infertility.^13^ AI blocks the aromatization of androgen to estrogen, and this has a similar effect to that of clomiphene citrate (CC), producing more follicles. Although the level of estrogen will decrease, AI does not have the same antiestrogen effects as CC. With this hypothesis in mind, this study aimed to retrospectively analyze whether a regimen of AI, to stimulate the growth of ovarian follicles, could reduce the occurrence of sERCs in oocytes.

## 2 MATERIALS AND METHODS

This was a retrospective cohort study of patients whose serum anti‐Müllerian hormone (AMH) levels were ≦1.0 ng/mL, involving a total of 467 single embryo transfer cycles (AI: 184 cycles, CC: 283 cycles) from 213 patients who underwent ICSI (AI: 103 patients, CC: 151 patients) between July 2014 and June 2016. Among these 213 patients, 62 patients (111 cycles) underwent only AI regimens, 110 patients (197 cycles) underwent only CC regimens, and 41 patients (159 cycles) underwent both AI and CC regimens. sERCs reoccurred 2‐3 times in five patients (2 with AI, 2 with CC and 1 with both AI and CC regimens). The clinical outcomes of patients undergoing frozen‐thawed single embryo transfers prior to the end of December 2017 were analyzed.

### 2.1 Stimulation protocols

The ovarian stimulation protocols were chosen depending on the patient's age and AMH level. Patients treated with AI and CC were in the same classification, and the choice of AI or CC was according to the patients' preferences; therefore, the two regimens, AI and CC, are comparable. With the CC protocol, 50 mg/day of clomiphene citrate (Clomid, Fuji Pharma, Tokyo, Japan) was administered once a day from cycle day 3 and continued up to the day of hCG administration. hMG (Folyrmon‐P, F, Fuji Pharma, Tokyo, Japan; HMG TEIZO, ASKA Pharmaceutical, Tokyo, Japan; Ferring, Ferring Pharmaceuticals, SA, Switzerland) was administered until the day of hCG administration. With the AI protocol, 5 mg/day of AI (Letrozole “TEVA”, Pharmaceutical Industries Ltd, Petah Tikva, Israel) was administered from cycle day 3 and continued for 5 days. hMG was administered until the day of hCG administration. When at least 2 follicles reached ≧18 mm in diameter, ovulation was induced with 10 000 IU recombinant human chorionic gonadotropin (HCG, Fuji Pharma, Toyama).

### 2.2 Timing of ICSI, detection of sERCs, and the cryopreservation of embryos

ICSI was performed on MII stage oocytes 4 hours after oocyte retrieval. MI stage oocytes were used for ICSI only when they reached MII stage maturity within 8 hours of oocyte retrieval. This approach was a consequence of reported data which demonstrated that there is no significant difference between the outcomes of MII oocytes and that of MI‐MII oocytes which reach MII on the day of oocyte retrieval.

Oocytes were classified morphologically into sERC‐positive and sERC‐negative oocytes at the time of ICSI under inverted microscopes (OLYMPUS, Tokyo, Japan at X400 magnification). The presence of sERCs was defined as being of a size sufficient to be observable by microscope and was generally more than 10µm in diameter. Sperm were prepared using a density‐gradient centrifugation technique with ISolate (Irvine, Cal., USA) and the swim‐up method with Universal IVF (Origio, Malov, Denmark). The day of cryopreservation depended entirely on blastocyst development. Blastocysts (grades 3 to 6) with good/ fair ICM‐TE morphology (score A‐B) were selectively cryopreserved on day 5. Blastocysts not reaching this benchmark were given one more day in culture and frozen on day 6 if they met the aforementioned criteria. Blastocyst cryopreservation was performed using vitrification media (Cryotop, KITAZATO, Shizuoka, Japan).

### 2.3 Statistical analysis

Statistical analysis was performed using the *χ* ^2^‐test or Student's t test where appropriate, employing JMP Software, Version 11 (SAS Institute Japan). Multiple logistic regression analysis was also used to obtain odds ratios in the presence of several explanatory variables. Differences were considered statistically significant when the *P*‐value was < .05.

## 3 RESULTS

The average age and AMH level were 41.4 ± 3.4 years and 0.35 ± 0.29 ng/mL, respectively, for patients treated with AI and 41.4 ± 3.5 years and 0.39 ± 0.29 ng/mL for those treated with CC. There were no statistical differences between the two regimens regarding age, AMH level, or the number of ICSI cycles (age: *P* = .981; AMH: *P* = .221; number of ICSI cycles: *P* = .991). The occurrence of sERCs was found to be 15.2% (28/184) in oocytes from patients treated with AI, which was significantly higher than that of 6.0% (17/283) in oocytes from those treated with CC (OR: 2.81, 95% CI: 1.49‐5.30, *P* = .001) (Table 1). Multiple logistic regression analysis was also used to obtain an odds ratio in the presence of several explanatory variables including age, serum progesterone (P4) levels, serum AMH levels, and number of ICSI attempts. Adjusting for these variables (OR: 2.83, 95% CI: 1.45‐5.45, *P* = .001) had no influence on the results. Table S1 shows the occurrence of sERCs in oocytes from patients treated with AI and CC, and compares the differences in patients when they were divided into two age categories (40> and 40≦). There were no statistical differences between the two regimens regarding age, AMH level, and the number of ICSI cycles. The occurrence of sERCs in oocytes from patients treated with AI was also significantly higher than that in oocytes from those treated with CC in each age category.

**Table 1:** The occurrence of sERCs in oocytes from patients treated with AI and CC

Regimen	AI	CC	*P*‐value
Patient's age (years)	41.4 ± 3.4	41.4 ± 3.5	.981
Serum AMH (ng/ml)	0.35 ± 0.29	0.39 ± 0.29	.221
N. of ICSI cycles (times)	5.6 ± 4.5	5.6 ± 4.4	.991
Occurrence of sERC (%)	15.2 (28/184)	6.0 (17/283)	.001

Note

Patient's age, level of serum AMH, and number of ICSI cycles are presented as means ± standard deviation.

Among the patients treated with AI, serum estradiol and progesterone levels on the date of hCG were 521.8 ± 324.7 pg/mL and 1.19 ± 0.68 ng/mL, respectively, in sERC (+) cycles. In sERC (−) cycles, they were 352.7 ± 283.5 pg/mL and 0.75 ± 0.50 ng/mL, respectively. Both estradiol and progesterone levels were significantly higher in sERC (+) cycles than in sERC (−) cycles (E2: *P* = .005; P4: *P* < .001). With regard to CC cycles, serum estradiol and progesterone levels on the date of hCG were 1024.2 ± 425.0 pg/mL and 0.82 ± 0.49 ng/mL in sERC (+) and 909.9 ± 444.7 pg/mL and 0.80 ± 0.47 ng/mL in sERC (−) cycles. As compared to the significant differences in serum estradiol and progesterone levels found in AI cycles, no significant differences were detected in CC cycles (E2: *P* = .304; P4: *P* = .826) (Table 2). The level of serum progesterone throughout the AI and CC regimens and the level of serum estradiol during the CC regimen remained constant. The level of estradiol during the AI regimen was not significantly different when patients were divided into two age categories (40 >and 40≦) (Table S2).

**Table 2:** Duration and total dosage of administration of stimulation drugs, serum estradiol and progesterone levels, and embryo development rates for each regimen in sERC (+) and sERC (−) cycles among patients treated with AI and CC

	sERC (+) cycles	sERC (−) cycles	*P*‐value
AI			
N. of the cases	24	91	
N. of the cycles	28	156	
Duration of AI (days)	5.0 ± 0.0	5.0 ± 0.0	1.000
Duration of hMG (days)	4.3 ± 1.9	3.0 ± 1.7	<.001
Total dosage of hMG (IU)	637.5 ± 284.7	452.9 ± 253.6	<.001
Daily dosage of hMG (IU)	150	150	
Serum estradiol level (pg/ml)	521.8 ± 324.7	352.7 ± 283.5	.005
Serum progesterone level (ng/ml)	1.19 ± 0.68	0.75 ± 0.50	<.001
N. of oocytes	71	407	
N. of MⅡoocytes	66	314	
2PN rate (%)	66.7 (44/66)	66.6 (209/314)	.987
1PN rate (%)	1.5 (1/66)	3.5 (11/314)	.361
>3PN rate (%)	10.6 (7/66)	2.2 (7/314)	.004
N. of embryos	40	158	
Blastocyst formation rate (%)	15.0 (6/40)	39.2 (62/158)	.002
High‐quality blastocyst formation rate (%)	5.0 (2/40)	13.9 (22/158)	.092
N. of transferred cycles	10	101	
Implantation rate	10.0% (1/10)	20.8% (21/101)	.380
Pregnancy loss rate	0% (0/1)	47.6% (10/21)	.263
Birth rate	10.0% (1/10)	10.9% (11/101)	.930
Congenital abnormality rate	0% (0/1)	0% (0/11)	
CC			
N. of the cases	14	145	
N. of the cycles	17	266	
Duration of CC (days)	10.4 ± 3.3	10.9 ± 3.4	.525
Duration of hMG (days)	2.9 ± 1.9	2.9 ± 1.4	.994
Total dosage of hMG (IU)	432.4 ± 285.0	432.0 ± 214.2	.994
Daily dosage of hMG (IU)	150	150	
Serum estradiol level (pg/ml)	1024.2 ± 425.0	909.9 ± 444.7	.304
Serum progesterone level (ng/ml)	0.82 ± 0.49	0.80 ± 0.47	.826
N. of oocytes	56	652	
N. of MⅡoocytes	48	544	
2PN rate (%)	64.6 (31/48)	71.3 (388/544)	.333
1PN rate (%)	4.2 (2/48)	2.0 (11/544)	.381
>3PN rate (%)	0 (0/48)	2.8 (15/544)	.109
N. of embryos	30	379	
Blastocyst formation rate (%)	36.7 (11/30)	42.2 (160/379)	.551
High‐quality blastocyst formation rate (%)	3.3 (1/30)	14.3 (54/379)	.051
N. of transferred cycles	7	122	
Implantation rate	28.6% (2/7)	22.1% (27/122)	.699
Pregnancy loss rate	100% (2*/2)	37.0% (10/27)	.053
Birth rate	0% (0/7)	13.9% (17/122)	.153
Congenital abnormality rate		0% (0/17)	

Note

Levels of serum estradiol and progesterone are presented as means ± standard deviation.

Duration of AI, CC, and hMG are presented as means ± standard deviation.

The duration of administration of AI in both sERC (+) and sERC (−) cycles was 5 days and that of CC in sERC (+) and sERC (−) cycles was 10.4 ± 3.3 days and 10.9 ± 3.4 days, respectively, which was not statistically different (*P* = .525). However, the duration and total dosage of hMG during the AI regimen in sERC (+) cycles were 4.3 ± 1.9 days and 637.5 ± 284.7 IU, respectively. Both of these figures were significantly different from those for sERC (−) cycles, with a duration of 3.0 ± 1.7 days (*P* < .001) and a dosage of 452.9 ± 253.6 IU (*P* < .001). The duration and total dosage of hMG during the CC regimen in sERC (+) cycles were 2.9 ± 1.9 days and 432.4 ± 285.0 IU. Neither of these figures was significantly different from the data for sERC (−) cycles, of 2.9 ± 1.4 days (*P* = .994) and 432.0 ± 214.2 IU (*P* = .994). 2PN and 1PN formation rates for the AI regimen in sERC (+) cycles were 66.7% (44/66) and 1.5% (1/66), respectively. Neither of these figures was significantly different from the data for sERC (−) cycles, of 66.6% (209/314) (OR: 1.00, 95% CI: 0.57‐1.76, *P* = .987) and 3.5% (11/314) (OR: 0.42, 95% CI:0.05‐3.34, *P* = .361) for 2PN and 1PN, respectively. The 3PN formation rate during the AI regimen was 10.6% (7/66) in sERC (+) cycles, which was significantly higher than that of sERC (−) cycles, at 2.2% (7/314) (OR: 5.20, 95% CI: 1.76‐15.38, *P* = .004). In contrast, no statistical difference was discovered during the CC regimen between the formation rate of sERC (+) cycles (0.0% (0/48) and that of 2.8% (15/544) in sERC (−) cycles) (*P* = .109). The blastocyst formation rate during the AI regimen in sERC (+) cycles (15.0% (6/40)) was also found to be significantly lower than that of 39.2% (62/158) (OR: 0.27, 95% CI: 0.11‐0.69, *P* = .002) during sERC (−) cycles. However, no statistical difference was detected for the CC regimen between 36.7% (11/30) for sERC (+) cycles and 42.2% (160/379) for sERC (−) cycles (OR: 0.79, 95% CI: 0.37‐1.71, *P* = .551). Regarding the high‐quality blastocyst formation rate, no statistical difference was discovered for either the AI or CC regimens (AI: 5.0% (2/40) with sERC (+) and 13.9% (22/158) sERC (−) cycles (OR: 0.33, 95% CI: 0.07‐1.45, *P* = .092), CC: 3.3% (1/30) for sERC (+) and 14.3% (54/379) in sERC (−) cycles (OR: 0.21, 95% CI: 0.03‐1.56, *P* = .051) (Table 2)). When the patients were separated into two age categories (40 >and 40≦), no significant difference was found between the AI and CC regimens among patients who were 40 or older. In contrast, among patients who were younger than 40, the duration of AI or CC administration and hMG administration was significantly longer for the AI regimen than CC (Table S3). Between sERC (+) and sERC (−) negative cycles for the AI and CC regimens, no significant differences were found regarding implantation, miscarriage or live birth rates or the occurrence rate of congenital abnormalities (Table S4). No significant differences in clinical outcomes were observed for either regimen when the patients were divided into two categories regarding their age (40> and 40≦) (Table S5). Please note, embryos derived from sERC (+) oocytes were only transferred when no embryos derived from sERC (−) oocytes were available for transfer.

## 4 DISCUSSION

The initial goal of this study was to reduce the occurrence of sERCs with the application of AI regimen, which has been reported to lower E2 levels. However, contrary to expectation, it was found that the occurrence of sERCs was significantly higher in oocytes from patients treated with AI than in those treated with CC. Furthermore, an increased occurrence of sERCs remained constant for the AI regimen when patients were divided into two age categories. This substantiates the hypothesis that the occurrence of sERCs is directly congruent with an elevation in progesterone. Considering the higher levels of progesterone in sERC (+) cycles, it may also be related to prolonged ovulation, with the negative effect of premature luteinization on oocytes. This may be explained by a previous report 14 that the occurrence of sERCs from AI with a GnRH antagonist regimen was no higher than that in other ovarian stimulation regimens, as administration of a GnRH antagonist suppresses premature P4 elevation. As premature luteinization frequently occurs with the process of AI without GnRH antagonist protocols, further study will be required to elucidate the causes of the occurrence of sERCs. It may also be beneficial to study whether sERC occurrence rate is higher in random‐start controlled ovarian stimulation when it is started at luteal phase with elevated progesterone. As the results of this study come from patients whose AMH level was less than or equal to 1.0 ng/mL, the occurrence of sERCs among patients with the presence of higher AMH levels is still unknown.

A further point of critical importance: the 3PN formation rate among embryos derived from sERC (+) oocytes was significantly higher than the 3PN formation rate of sERC (−) oocytes. This was consistent with previous findings, that the rate of 3PN formation is higher in sERC (+) than sERC (−) oocytes.^9^ As a failure of the second polar body extrusion results in 3PN formation, and mitotic failure has also been reported to be higher in embryos derived from sERC (+) oocytes, the transfer of embryos derived from sERC (+) oocytes should be performed with caution. Furthermore, a recent paper found that the presence of sERCs in oocytes alters the molecular structure. This has the potential to cause mitotic and meiotic errors and negatively affect the spindle assembly, organization of cytoskeleton, the microtubules, and the mitochondrial structure and activity.^15^

sERCs usually appear in MII stage oocytes rather than in MI and GV stage oocytes and also appear when the metaphase stage is extended.^9^ It has also been suggested that the occurrence of sERCs could also be a sign of prolonged cytoplasmic maturation prior to the triggering of LH surges in controlled ovarian stimulation cycles.^1^, ^9^ As a significantly higher occurrence of sERCs was found in oocytes from patients treated with AI regimen, despite lower levels of serum estradiol, the elevation of estradiol itself is unlikely to be the cause of sERC formation and probably the consequence of other factors such as an extended MII stage. Supplemental videos provided by Otsuki et al showed that sERCs appeared during mitosis after prolonged periods of metaphase and in oocytes when the 2PN stage was overextended.^9^ The reasons behind ER aggregation are still unknown; however, it is thought that it could be related to aberrant timings of calcium release. Although sERCs are usually surrounded by mitochondria,^1^ their relationship with other organelle has not yet been discovered. As the size of sERCs increases during the culture of unfertilized oocytes and sERCs move within the cytoplasm to form larger clusters,^1^, ^9^ sERCs may be associated with specific organelles within oocytes.

This study also found that the duration of AI and hMG administration was longer for sERC (+) than for sERC (−) cycles. Additionally, the duration of administration was longer for AI than for CC regimens. Thus, the occurrence of sERCs could be unrelated to estradiol levels. As the half‐life of letrozole is shorter than that of CC,^16^ the influence of letrozole should be less significant than that of CC. There was no significant difference in clinical outcomes between transferred embryos derived from sERC (+) or sERC (−) oocytes. Moreover, no significant difference was detected between the AI or CC regimens, even when patients were separated into two age categories (40> and 40≦).

In this study, comparison was limited to the two aforementioned ovarian stimulation protocols. Other stimulation protocols, such as long, short, and antagonist protocols, could not be compared with AI in this study, as the patients' backgrounds (age and levels of AMH) varied too greatly. However, our results provide an important insight for future studies investigating the mechanism underlying sERC formation.

## CONFLICTS OF INTEREST

Hiroe Saito, Junko Otsuki, Hiromi Takahashi, Rei Hirata, Toshihiro Habara and Nobuyoshi Hayashi declare that they have no conflicts of interest.

## HUMAN/ANIMAL RIGHTS

This article does not contain any experimental studies with human or animal subjects performed by any of the authors. This study received the approval of the institutional review board of Okayama Couple's Clinic (Approval number: 18000128‐12).
