# Supplementary material for: A higher incidence of smooth endoplasmic reticulum clusters with aromatase inhibitors
Source: Reprod Med Biol. 2019 Sep 11;18(4):384–9. doi: 10.1002/rmb2.12296 (PMC6780026; doi:10.1002/rmb2.12296)
Supplement: Supplementary file 1 [file RMB2-18-384-s001.docx]

Supplementary Table1

The occurrence of sERCs in oocytes from patients treated with AI and CC, and the differences in patients when they were divided into two age categories (40> and 40≦)

| Regimen | AI | CC | *P*-value |
| --- | --- | --- | --- |
| <40 |  |  |  |
| Patient’s age (years) | 37.0±2.1 | 36.5±2.2 | 0.177 |
| Serum AMH (ng/ml) | 0.34±0.28 | 0.33±0.25 | 0.840 |
| N. of ICSI cycles (times) | 4.8±3.5 | 4.1±3.4 | 0.286 |
| Occurrence of sERC (%) | 17.0 (9/53) | 4.2 (3/72) | 0.016 |
| ≧40 |  |  |  |
| Patient’s age (years) | 43.2±2.0 | 43.0±1.9 | 0.623 |
| Serum AMH (ng/ml) | 0.35±0.29 | 0.41±0.30 | 0.152 |
| N. of ICSI cycles (times) | 6.0±4.8 | 6.2±4.6 | 0.747 |
| Occurrence of sERC (%) | 14.5 (19/131) | 6.6 (14/211) | 0.018 |

Patient’s age, level of serum AMH and number of ICSI cycles are presented as means ± standard deviation.
