# Supplementary material for: A higher incidence of smooth endoplasmic reticulum clusters with aromatase inhibitors
Source: Reprod Med Biol. 2019 Sep 11;18(4):384–9. doi: 10.1002/rmb2.12296 (PMC6780026; doi:10.1002/rmb2.12296)
Supplement: Supplementary file 2 [file RMB2-18-384-s002.docx]

Supplementary Table 2

Serum estradiol and progesterone levels in sERC (+) and sERC (-) cycles among patients treated with AI and CC when the patients were divided into

two categories regarding their age (40> and 40≦)

| Regimen | | sERC (+) cycles | sERC (-) cycles | *P*-value |
| --- | --- | --- | --- | --- |
| <40 |  |  |  |  |
| AI | Serum estradiol level (pg/ml) | 498.4±298.7 | 332.4±269.5 | 0.104 |
| AI | Serum progesterone level (ng/ml) | 1.22±0.55 | 0.75±0.42 | 0.006 |
| CC | Serum estradiol level (pg/ml) | 1255.7±321.5 | 895.6±456.3 | 0.182 |
| CC | Serum progesterone level (ng/ml) | 1.10±0.10 | 0.79±0.40 | 0.184 |
| ≧40 |  |  |  |  |
| AI | Serum estradiol level (pg/ml) | 532.9±343.6 | 360.6±289.6 | 0.021 |
| AI | Serum progesterone level (ng/ml) | 1.17±0.75 | 0.74±0.53 | 0.003 |
| CC | Serum estradiol level (pg/ml) | 974.6±437.5 | 914.9±441.7 | 0.626 |
| CC | Serum progesterone level (ng/ml) | 0.76±0.52 | 0.80±0.50 | 0.788 |

Levels of serum estradiol and progesterone are presented as means ± standard deviation.
