# Supplementary material for: A higher incidence of smooth endoplasmic reticulum clusters with aromatase inhibitors
Source: Reprod Med Biol. 2019 Sep 11;18(4):384–9. doi: 10.1002/rmb2.12296 (PMC6780026; doi:10.1002/rmb2.12296)
Supplement: Supplementary file 3 [file RMB2-18-384-s003.docx]

Supplementary Table 3

Total dosage and duration of administration of stimulation drugs and embryo development rates for each regimen when the patients were divided into two categories regarding their age (40> and 40≦)

| Regimen | AI | CC | *P*-value |
| --- | --- | --- | --- |
| <40 |  |  |  |
| Duration of AI or CC (days) | 5 | 11.4±4.6 |  |
| Duration of hMG (days) | 3.7±1.8 | 2.8±1.6 | 0.005 |
| Total dosage of hMG (IU) | 551.9±272.4 | 420.8±235.7 | 0.005 |
| Daily dosage of hMG (IU) | 150 | 150 |  |
| N. of oocytes | 149 | 177 |  |
| N. of MⅡoocytes | 115 | 151 |  |
| 2PN rate (%) | 71.3 (82/115) | 70.2 (106/151) | 0.844 |
| 1PN rate (%) | 5.2 (6/115) | 4.6 (7/151) | 0.828 |
| >3PN rate (%) | 3.5 (4/115) | 2.0 (3/151) | 0.454 |
| N. of embryos | 65 | 99 |  |
| Blastocyst formation rate (%) | 43.1 (28/65) | 58.6 (58/99) | 0.052 |
| High quality blastocyst  formation rate (%) | 20.0 (13/65) | 26.3 (26/99) | 0.353 |
| ≧40 |  |  |  |
| Duration of AI or CC (days) | 5 | 10.7±2.8 |  |
| Duration of hMG (days) | 3.0±1.7 | 2.9±1.4 | 0.522 |
| Total dosage of hMG (IU) | 452.3±259.1 | 435.8±212.7 | 0.522 |
| Daily dosage of hMG (IU) | 150 | 150 |  |
| N. of oocytes | 329 | 531 |  |
| N. of MⅡoocytes | 265 | 441 |  |
| 2PN rate (%) | 64.5 (171/265) | 71.0 (313/441) | 0.075 |
| 1PN rate (%) | 2.3 (6/265) | 1.4 (6/441) | 0.376 |
| >3PN rate (%) | 3.8 (10/265) | 2.7 (12/441) | 0.441 |
| N. of embryos | 133 | 310 |  |
| Blastocyst formation rate (%) | 30.1 (40/133) | 36.5 (113/310) | 0.193 |
| High quality blastocyst  formation rate (%) | 8.3 (11/133) | 9.4 (29/310) | 0.713 |

Duration of AI, CC and hMG are presented as means ± standard deviation.
