# Supplementary material for: A higher incidence of smooth endoplasmic reticulum clusters with aromatase inhibitors
Source: Reprod Med Biol. 2019 Sep 11;18(4):384–9. doi: 10.1002/rmb2.12296 (PMC6780026; doi:10.1002/rmb2.12296)
Supplement: Supplementary file 4 [file RMB2-18-384-s004.doc]

Supplementary Table 4

Clinical outcomes of AI and CC regimens for each fresh and frozen cycle

|  |  | sERC (+) | sERC (-) | *P*-value |
| --- | --- | --- | --- | --- |
| AI |  |  |  |  |
| N. of transferred  cycles | Fresh | 5 | 58 |  |
|  | Frozen | 5 | 43 |  |
|  | Total | 10 | 101 |  |
| Implantation rate | Fresh | 0% (0/5) | 19.0% (11/58) | 0.157 |
|  | Frozen | 20.0% (1/5) | 23.3% (10/43) | 0.868 |
|  | Total | 10.0% (1/10) | 20.8% (21/101) | 0.380 |
| Pregnancy loss  rate | Fresh | - | 36.4% (4/11) |  |
|  | Frozen | 0% (0/1) | 60.0% (6/10) | 0.192 |
|  | Total | 0% (0/1) | 47.6% (10/21) | 0.263 |
| Birth rate | Fresh | 0% (0/5) | 12.1% (7/58) | 0.267 |
|  | Frozen | 20.0% (1/5) | 9.3% (4/43) | 0.498 |
|  | Total | 10.0% (1/10) | 10.9% (11/101) | 0.930 |
| Congenital abnormality rate | Fresh | - | 0% (0/7) |  |
|  | Frozen | 0% (0/1) | 0% (0/4) |  |
|  | Total | 0% (0/1) | 0% (0/11) |  |
| CC |  |  |  |  |
| N. of transferred  cycles | Fresh | 1 | 8 |  |
|  | Frozen | 6 | 114 |  |
|  | Total | 7 | 122 |  |
| Implantation rate | Fresh | 0% (0/1) | 12.5% (1/8) | 0.617 |
|  | Frozen | 33.3% (2/6) | 22.8% (26/114) | 0.568 |
|  | Total | 28.6% (2/7) | 22.1% (27/122) |  |
| Pregnancy loss  rate | Fresh | - | 100% (1/1) |  |
|  | Frozen | 100% (2*/2) | 38.5% (10/26) | 0.058 |
|  | Total | 100% (2*/2) | 37.0% (10/27) | 0.053 |
| Birth rate | Fresh | 0% (0/1) | 12.5% (1/8) | 0.617 |
|  | Frozen | 0% (0/6) | 14.0% (16/114) | 0.184 |
|  | Total | 0% (0/7) | 13.9% (17/122) | 0.153 |
| Congenital abnormality rate | Fresh | - |  |  |
|  | Frozen | - | 0% (0/16) |  |
|  | Total | - | 0% (0/17) |  |

*One stillbirth, occurring at 24 weeks and 6 days has been included.

Note that embryos derived from sERC (+) oocytes were only transferred when no embryos derived from sERC (-) oocytes were available for transfer.
