# Supplementary material for: A higher incidence of smooth endoplasmic reticulum clusters with aromatase inhibitors
Source: Reprod Med Biol. 2019 Sep 11;18(4):384–9. doi: 10.1002/rmb2.12296 (PMC6780026; doi:10.1002/rmb2.12296)
Supplement: Supplementary file 5 [file RMB2-18-384-s005.doc]

Supplementary Table 5

Clinical Outcomes of AI and CC regimes for each fresh and frozen cycle when the patients were divided into two categories regarding their age (40> and 40≦)

| Regimen |  | AI | CC | *P*-value |
| --- | --- | --- | --- | --- |
| <40 |  |  |  |  |
| N. of transferred  cycles | Fresh | 21 | 6 |  |
|  | Frozen | 24 | 44 |  |
|  | Total | 45 | 50 |  |
| Implantation rate | Fresh | 28.6% (6/21) | 16.7% (1/6) | 0.544 |
|  | Frozen | 37.5% (9/24) | 45.5% (20/44) | 0.525 |
|  | Total | 33.3% (15/45) | 42.0% (21/50) | 0.384 |
| Pregnancy loss  rate | Fresh | 16.7% (1/6) | 0% (0/1) | 0.563 |
|  | Frozen | 66.7% (6/9) | 35.0% (7*/20) | 0.111 |
|  | Total | 46.7% (7/15) | 33.3% (7*/21) | 0.419 |
| Birth rate | Fresh | 23.8% (5/21) | 16.7% (1/6) | 0.704 |
|  | Frozen | 12.5% (3/24) | 29.6% (13/44) | 0.100 |
|  | Total | 17.8% (8/45) | 28.0% (14/50) | 0.235 |
| Congenital abnormality rate | Fresh | 0% (0/5) | 0% (0/1) |  |
|  | Frozen | 0% (0/3) | 0% (0/13) |  |
|  | Total | 0% (0/8) | 0% (0/14) |  |
| ≧40 |  |  |  |  |
| N. of transferred  cycles | Fresh | 42 | 3 |  |
|  | Frozen | 24 | 76 |  |
|  | Total | 66 | 79 |  |
| Implantation rate | Fresh | 11.9% (5/42) | 0% (0/3) | 0.392 |
|  | Frozen | 8.3% (2/24) | 10.5% (8/76) | 0.751 |
|  | Total | 10.6% (7/66) | 10.1% (8/79) | 0.925 |
| Pregnancy loss  rate | Fresh | 60.0% (3/5) | - |  |
|  | Frozen | 0% (0/2) | 62.5% (5/8) | 0.070 |
|  | Total | 42.9% (3/7) | 62.5% (5/8) | 0.446 |
| Birth rate | Fresh | 4.8% (2/42) | 0% (0/3) | 0.595 |
|  | Frozen | 8.3% (2/24) | 4.0% (3/76) | 0.416 |
|  | Total | 6.1% (4/66) | 3.8% (3/79) | 0.528 |
| Congenital abnormality rate | Fresh | 0% (0/2) | - |  |
|  | Frozen | 0% (0/2) | 0% (0/3) |  |
|  | Total | 0% (0/4) | 0% (0/3) |  |

*One stillbirth, occurring at 24 weeks and 6 days has been included.

Note that embryos derived from sERC (+) oocytes were only transferred when no embryos derived from sERC (-) oocytes were available for transfer.
